# Accuracy of Toric Intraocular Lens Implantation Using Three‐Random‐Point Marking Method: A Prospective Pilot Study

**DOI:** 10.1155/joph/7948866

**Published:** 2026-04-27

**Authors:** Shuilian Chen, Yiliang Liu, Fulong Luo, Hongyang Zhang, Yongyi Niu

**Affiliations:** ^1^ Department of Ophthalmology, Guangdong Provincial People’s Hospital, Guangdong Academy of Medical Sciences, Southern Medical University, Guangzhou, China, fimmu.com; ^2^ Guangdong Cardiovascular Institute, Guangdong Provincial People’s Hospital, Guangdong Academy of Medical Sciences, Guangzhou, China, gdghospital.org.cn; ^3^ Department of Ophthalmology, Nanfang Hospital, Southern Medical University, Guangzhou, China, fimmu.com

**Keywords:** axis alignment, cataract, three-random-point marking, toric intraocular lens

## Abstract

**Introduction:**

This study aimed to compare the accuracy of three‐random‐point (TRP) marking method with the slit‐lamp horizontal meridian (SHM) marking method by using an image‐guided system in the positioning of a toric intraocular lens (IOL).

**Materials and Methods:**

A total of 60 eyes of 54 patients who underwent cataract surgery were prospectively randomized into 3 groups (each group included 20 eyes: Group 1, novice doctor and dilated pupil patient; Group 2, novice doctor and small pupil patient; and Group 3, experienced doctor and small pupil patient). Every patient was marked three points (horizontal two points and random third point) in corneal limbus with a staining blunt needle and then measured by the iTrace system. The axis deviations from the SHM method (axis A, horizontal two points method) and the TRP method (axis B, three points method) were measured by the digital image‐guided navigation system.

**Results:**

The axis deviations of each group showed a significant difference between the SHM and TRP marking methods (Group 1: SHM method: 3.05° ± 2.58° and TRP method: 0.85° ± 0.93°, *p* < 0.01; Group 2: SHM method: 2.15° ± 2.35° and TRP method: 1.05° ± 1.57°, *p* < 0.05; and Group 3: SHM method: 1.00° ± 1.84° and TRP method: 0.45° ± 1.23°, *p* < 0.05).

**Conclusions:**

The TRP marking method using the iTrace aberrometer was found to be more effective and more accurate than SHM marking method in the positioning of toric IOLs before surgery.

**Trial Registration:** Chinese Clinical Trial Registry: ChiCTR2600121114

## 1. Introduction

Corneal astigmatism is frequently found in cataract patients. Studies show about 86.6% of them have preoperative astigmatism; among these, 40% exhibit about > 1.0 D and 20% have over > 1.5 D [1, 2]. Residual astigmatism after cataract surgery affects the patient’s postoperative visual acuity, and it is now generally accepted by surgeons that corneal astigmatism > 0.75 D needs to be corrected during cataract surgery [3]. The main methods of astigmatism correction commonly used include limbal relaxing incisions, peripheral corneal relaxing incisions, toric intraocular lens (IOL) implantation, and excimer laser surgery [4]. Among them, the implantation of toric IOL and corneal relaxing incisions are commonly used for astigmatism correction in cataract surgery [5]. Toric IOL implantation during cataract surgery was more widely used owing to its reliability and effectiveness. However, inaccurate intraoperative toric IOL implantation and early postoperative toric IOL rotation are the main risk factors for astigmatic undercorrection in patients after cataract surgery. Specifically, accurate preoperative marking is the most important prerequisite to ensure the accuracy of intraoperative toric IOL implantation axis [6]. Currently, manual marking methods mainly include slit‐lamp marking, surgeon’s direct visual marking, bubble marker–assisted method, pendular marker–assisted method, and tonometer marking, which are widely applicated in clinical practice [7]. Nevertheless, these traditional marking methods are largely dependent upon the surgeon’s level and the degree of patient’s cooperation [8].

In recent years, the digital image navigation during cataract surgery has been confirmed to be more accurate for the actual axis ascertain than manual marking methods mentioned above [9]. Moreover, the digital image navigation serves as the gold standard for the IOL alignment [10], whereas it demands expensive equipment and intraoperative microscope integration with complex image registration. While femtosecond laser–based marking methods also demonstrate good precision, they rely on high‐cost, advanced femtosecond laser platforms with iris registration [11]. Therefore, it is difficult for the digital image navigation and femtosecond laser–based marking methods to be widely applied in hospitals at all levels and a new method to marking IOL axis is urgently required so far. According to our previous research [7], we compared the three‐random‐point (TRP) and the slit‐lamp horizontal meridian (SHM) marking methods and proved that TRP was a new and effective method of preoperative limbal marking in IOL alignment. The TRP marking method merely needs three random points on the corneal limbus, making it a more universally accessible and cost‐effective solution for surgical centers, as it does not require intraoperative microscope integration or complex image registration. It eliminates the axis misalignment resulting from varying head positions and could locate the Wallace Mendez Degree Gauge during surgery with the three points. However, the difference between TRP and SHM has not been ascertained before. Additionally, the accuracy evaluation of the TRP marking method was determined using the iTrace aberrometer system, which differed from patient’s head position. Further studies are required to determine whether the TRP labeling method can be well applied in clinical practice.

Consequently, given that digital image navigation is the gold standard for IOL alignment, this study uniquely utilizes this modality to conduct a direct and granular comparison between the TRP and SHM marking methods. Unlike previous studies, including our own preliminary work [7], we specifically evaluate the clinical robustness of the TRP method by assessing its accuracy across different levels of surgical expertise (novice vs. experienced) and under challenging condition (dilated pupil). This comprehensive validation addresses critical gaps in the existing literature and aims to establish TRP as a reliable and practical alternative for toric IOL alignment.

## 2. Materials and Methods

### 2.1. Study Design, Ethical Approval, and Participants

This three‐arm, single‐center, randomized, double‐blind trial was conducted between September 2023 and November 2023 at Guangdong Eye Institute, Department of Ophthalmology, Guangdong Provincial People’s Hospital. This study protocol was reviewed and approved by the Medical Research Ethics Committee (KY‐Z2021‐041‐02 and KY2025‐954‐01) of Guangdong Provincial People’s Hospital (Guangdong Academy of Medical Sciences) and was conducted in accordance with the principles of the Declaration of Helsinki. All patients admitted for elective cataract surgery were screened for eligibility. The inclusion criteria were elective cataract surgery, age range between 50 and 90 years old at the time of enrollment, and provision of a signed informed consent form. The exclusion criteria were patients with previous intraocular surgery, corneal opacities, irregular astigmatism, glaucoma, macular disease, or diabetic retinopathy, patients who were unable to cooperate with the ophthalmological examination or complete the follow‐up, and patients who developed other serious systemic diseases during the follow‐up period that could affect the results.

### 2.2. Sample Size Calculation, Randomization, and Masking

As this study is a pilot study, there is no need to perform efficacy calculations to determine the sample size. Considering the standards and guidelines of the pilot study, we set the sample size to 50 participants [12, 13]. According to the expectation of a 5% dropout rate, the final sample size was set to 54 participants (6 underwent bilateral cataract surgery and 48 underwent unilateral cataract surgery, 60 eyes, 20 per arm) [14].

Randomization was performed using computerized random number tables. After provision of informed consent, all patients were randomly divided into the novice doctor (40 eyes: 20 eyes were nondilated pupils and 20 eyes were dilated pupils) and experienced doctor marking group (20 eyes: small pupils). Novice doctor was who had never performed axial marking on any patients’ eyes, whereas the experienced doctor was characterized as who had conducted axial marking on more than 100 individuals. Table 1 shows the overview of the included groups. The patient and treating physicians and nurses were blinded to the study intervention. An unblinded investigator performed the randomization before preoperative marking.

**TABLE 1:** Overview of the included groups.

Marking method	Group 1	Group 2	Group 3
Novice doctor	Yes	Yes	
Experienced doctor			Yes
Small pupil		Yes	Yes
Dilated pupil	Yes		

### 2.3. Preoperative Procedures

We compare the difference of target axis through TRP and SHM marking methods on the same eye. After topical anesthesia was administered, three limbal points were marked with a blunt staining needle on an upright patient. With the slit‐lamp (Haag‐Streit AG) set to a horizontal slit, the beam was focused on the corneal apex to guide the marking of the horizontal meridian at the limbus. The third point was then randomly marked. The two points located on the horizontal meridian were utilized as markers for the SHM marking method, while three points (including a randomly selected third point) were employed for the TRP marking methods. Corneal topography and anterior segment imaging were performed using the iTrace system (Tracey Technologies Corp., TX, USA). A Zaldivar Toric Caliper was then employed to connect the corneal center to the premarked limbal points (Figure 1(a)), allowing the meridian values to be directly read from the caliper.

FIGURE 1Representative photographs of axis alignment (the same individual). (a) Three blue points were marked in the corneal limbus. Points 4° and 184°/4° served as horizontal meridian (0°–180°) in SHM marking, plus the point 324°/144° as TRP marking. (b) Callisto eye system showed the actual horizontal meridian (yellow dashed line, 0°–180°) and the target axis (Z align, 3 parallel red lines, 120°). (c) In the SHM marking group, the corresponding target axis A (yellow triangle, 120°) was marked based on the horizontal meridian. Light blue dashed line represented the actual target axis A (Z align). (d) In the TRP marking group, the corresponding target axis B (yellow triangle, 120°) was marked based on the three points (4°, 184°/4° and 324°/144°). Light blue dashed line represented the actual target axis B (Z align).

**Figure:**
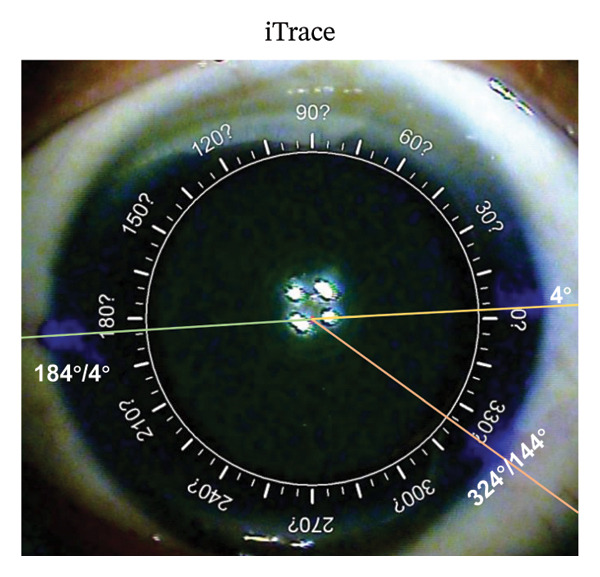
(a)

**Figure:**
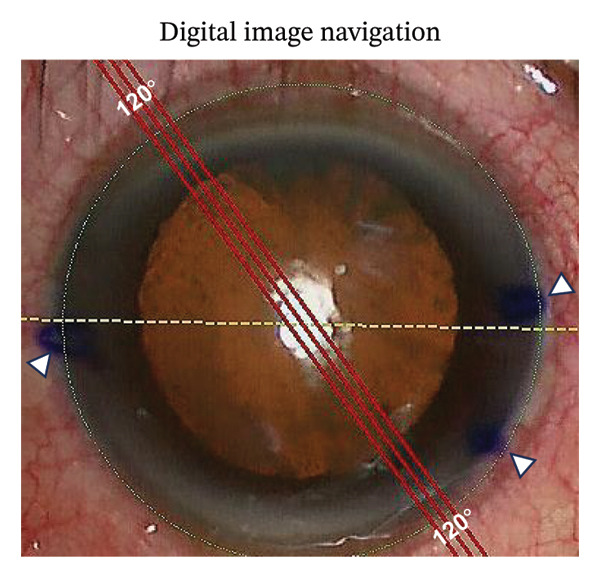
(b)

**Figure:**
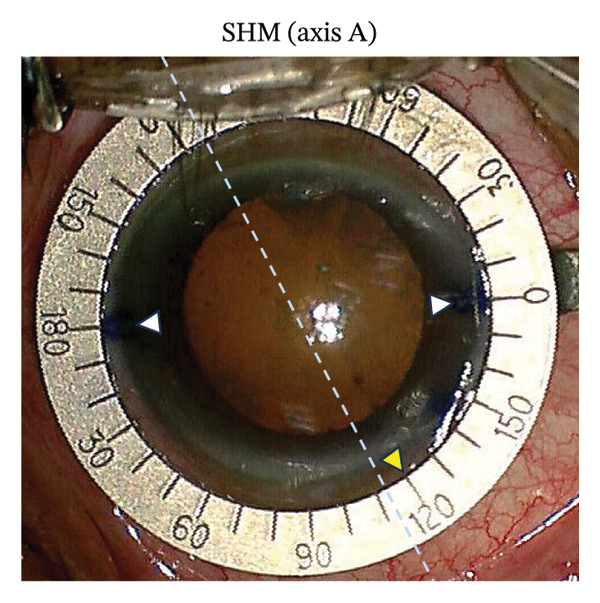
(c)

**Figure:**
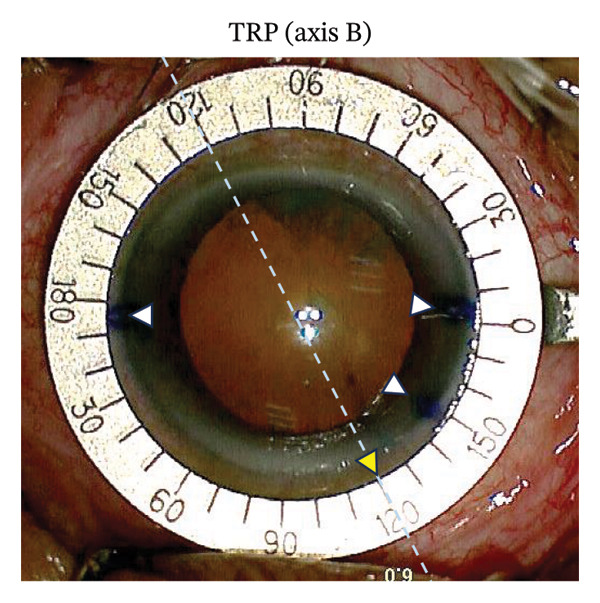
(d)

### 2.4. Intraoperative Procedures

We performed the procedure in the Callisto eye group by first capturing a reference image with the IOLMaster 700 and transferring the data to the Callisto eye system, which was linked to an OPMI Lumera 700 surgical microscope. The system then captured live microscope images and registered them, overlaying a translucent guide. We confirmed correct registration by visualizing the limbal vessels. Finally, the system displayed the planning graphics: three parallel red lines (Z‐align) for the target axis and a yellow dashed line marking the 0°–180° axis (Figure 1(b)).

During cataract surgery, toric or nontoric IOL was implanted. Alignment axis of the IOL was obtained with the Calculation Tool software (Version 3.2.4) provided on the Alcon website (https://www.acrysoftoriccalculator.com). The Wallace Mendez Degree Gauge (Bausch & Lomb, Inc.) was placed at the marked points based on the Zaldivar Toric Caliper measurement (Figures 1(c) and 1(d)). To streamline data collection and enhance statistical comparability, the astigmatic axis of implanted nontoric IOL was uniformly set at 120° using the image‐guided system to determine the actual axis marked by TRP and SHM methods.

### 2.5. Assessment of Misalignment

Axis misalignment was evaluated based on the difference between the target axis and the actual axis and measured using the Zaldivar Toric Caliper and Z align in the image‐guided system.

In the SHM marking group, the slit‐lamp position of 0°–180° was marked (Fazio; Janach, Como, Italy) and the corresponding target axis A (toric IOL: target axis; nontoric IOL: 120°) was marked in the limbus by scratching the cornea with a needle (Target axis A, yellow triangle). Similarly, target axis B (yellow triangle) was marked in the TRP marking group. Then, the actual axes A and B were measured by the 3 parallel red lines. However, the 3 parallel red lines could not be captured due to the reflection of Wallace Mendez Degree Gauge. Therefore, we use the blue dashed line to represent Z align in Figures 1(c) and 1(d).

### 2.6. Statistical Analysis

The study data were analyzed using SPSS Statistics, Version 19.0 (IBM Corp., Armonk, NY, USA). Values are expressed as mean ± standard deviation (SD). Student’s *t* test was used for comparison of between group variables. Normality of the data distribution was tested by the Shapiro–Wilk test. Continuous data were shown as the mean ± SD or median with interquartile range. Group comparisons between continuous variables were performed using the Student’s *t*‐test for parametric variables or Mann–Whitney *U* test for nonparametric variables. A *p* value of < 0.05 was considered statistically significant.

## 3. Results

### 3.1. Baseline Characteristic

This study included 60 eyes of 54 patients undergoing cataract surgery with implantation of an IOL (toric and nontoric). Group 1 included 8 women and 11 men, with a mean age of 70.95 ± 8.38 (range: 53–88 years). Group 2 included 9 women and 10 men, with a mean age of 70.05 ± 9.03 (range: 51–82 years). Group 3 included 9 women and 11 men, with a mean age of 67.10 ± 9.90 (range: 49–84 years). All eyes underwent surgery with both marking methods (SHM and TRP) without intraoperative complications. Table 2 shows the demographic characteristics of preoperative patient.

**TABLE 2:** Baseline demographic characteristics (preoperative).

Characteristic	Group 1	Group 2	Group 3
Age (years), mean ± SD	70.95 ± 8.38	70.05 ± 9.03	67.10 ± 9.90
Sex (F/M)	42%/58%	47%/53%	45%/55%
Right/left eye	45%/55%	45%/55%	50%/50%
Uncorrected visual acuity (logMAR)	0.58 ± 0.72	0.63 ± 0.60	0.42 ± 0.53
Best corrected visual acuity (logMAR)	0.37 ± 0.61	0.39 ± 0.54	0.27 ± 0.47
Axial length (mm)	24.38 ± 1.82	24.07 ± 2.47	24.15 ± 2.64

Abbreviation: SD, standard deviation.

### 3.2. TRP Method Was More Precise for Marking Axis Than SHM Method

**FIGURE 2:**
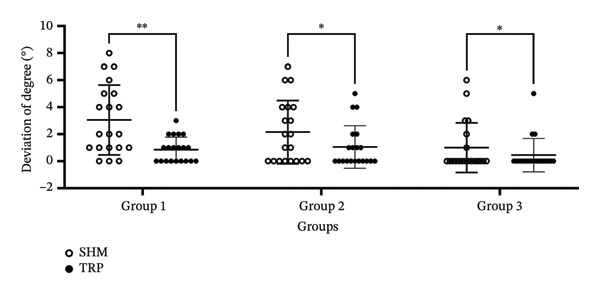
Mean axis deviation of each group. Hollow circle represented the SHM group and solid circle represented the TRP group. Group details are shown in Tables 1 and 3. ^∗∗^ *p* < 0.01^∗^ *p* < 0.05.

In order to further study the effect of ophthalmologists’ proficiency and whether the dilated pupil influence the marking results, the patients were divided into three groups randomly: Group 1 (novice doctor with dilated pupil patient), Group 2 (novice doctor with small pupil patient), and Group 3 (experienced doctor with small pupil patient). As shown in Figure 2 and Table 3, the mean misalignment was 3.05° ± 2.58° (range: 0°–9°), 2.15° ± 2.35° (range: 0°–7°), and 1.00° ± 1.84° (range: 0°–6°) for the SHM marking method in Group 1, Group 2, and Group 3, respectively. Misalignment of the TRP marking method was 0.85° ± 0.93° (range: 0°–3°), 1.05° ± 1.57° (range: 0°–5°), and 0.45° ± 1.23° (range: 0°–5°) in Group 1, Group 2, and Group 3, respectively. We found that regardless of each group, whether the doctor is novice or experienced, or whether the pupil is dilated or not, the axis deviation of the TRP method is significantly lower than that of the SHM method using the Callisto eye image‐guided system as the gold standard of target axis, which further confirmed our previous study [7].

**TABLE 3:** Results of axis deviation of each group.

Groups	SHM marking group	TRP marking group	*p* value^∗^
Group 1	3.05° ± 2.58°	0.85° ± 0.93°	0.0005
Group 2	2.15° ± 2.35°	1.05° ± 1.57°	0.0189
Group 3	1.00° ± 1.84°	0.45° ± 1.23°	0.0235

Abbreviations: SHM, slit‐lamp horizontal meridian; TRP, three random points.

^∗^ *p* < 0.05.

### 3.3. The Impact of Physician Experience and Patient Pupil Size on TRP and SHM Methods

However, there is no significant difference between novice and experienced doctor in the SHM or TRP method (SHM method: novice doctor: 2.15° ± 2.35° and experienced doctor: 1.00° ± 1.84°, *p* = 0.13; TRP method: novice doctor: 1.05° ± 1.57° and experienced doctor: 0.45° ± 1.23°, *p* = 0.20). Specially, in the TRP method, the misalignment in dilated pupil patient was 0.2° less than that in the small pupil patient, while in the SHM method, the misalignment in dilated pupil patient was 0.9° more than that in the small pupil patient although there was no statistical difference between dilated and small pupil eyes in the SHM or TRP method. This might be attributed to the small sample size (SHM method: novice doctor: 2.15° ± 2.35° and experienced doctor: 3.05° ± 2.58°, *p* = 0.24; TRP method: novice doctor: 1.05° ± 1.57° and experienced doctor: 0.85° ± 0.93°, *p* = 0.61).

## 4. Discussion

Preoperative axis determination and accurate intraoperative axial alignment of the modality (arcuate keratotomies and toric IOLs) utilized to correct corneal astigmatism are crucial in order to achieve optimal refractive outcomes. In this study, we proposed using the TRP method for marking axis to improve the convenience and accuracy and conducted clinical verification in the previous study [7]. On this basis, this study utilized the digital image navigation system as the gold standard and analyzed the accuracy of the two methods for novice and experienced doctors, when the patient’s pupils are dilated or not, respectively. We found that the TRP method was more stable than the SHM method regardless of physician proficiency and patient pupil status.

In the novice doctor group, the difference in axis misalignment between the two methods was 1.10°, while in the experienced doctor group, it was only 0.55°. Experienced doctors themselves have higher marking stability, which is in line with our expectations. The insignificant difference might be attributed to the small sample size. Similarly, the difference between the two methods of axis misalignment in the nonmydriasis group was 1.10° while that in the mydriasis group was 2.2°. This is also consistent with our expectations. Specifically, when the pupils are in a dilated status, ascertaining the precise horizontal misalignment of the pupil becomes inherently challenging due to the altered anatomy and optical properties of the eye under such conditions. This inherent difficulty poses a significant impediment to the conventional horizontal marking technique, which relies on the accurate determination of the pupil’s position to ensure the validity of subsequent interventions. Consequently, it is advisable to avoid conducting a dilated pupil when performing horizontal marking on the patient. In such scenarios, the TRP method that is less sensitive to the pupil size becomes particularly advantageous. Our data indicated that the TRP method had an axial error of only 0.85° in the case of mydriasis whereas that of the SHM method was 3.05°. Indeed, rotations within 10°—whether from imprecise initial placement, postoperative displacement, or a combination—might have little clinical impact. However, the accuracy of preoperative marking directly determines the precision of the starting point. For example, an initial error of 3–5° combined with 3–5° of postoperative rotation could easily reach or exceed the 10° clinical threshold. For astigmatic multifocal IOLs, even small residual astigmatism could affect outcomes, making precise marking especially critical. While minor deviations might have limited clinical effect, improved marking accuracy is likely beneficial.

Toric IOL axis marking methods are essential for accurate alignment of the IOL during cataract surgery to correct astigmatism. There are various methods for marking the target axis, including manual marking with a surgical marker, digital alignment systems, femtosecond laser–assisted capsular marks, and intraoperative aberrometry [15]. Advanced digital systems like the Verion (Alcon), Callisto (Zeiss), and iTrace use high‐definition preoperative images linked to keratometric data. These systems can display the intended axis for IOL alignment on a graphic overlay or guide femtosecond laser marking of the cornea with intrastromal corneal cuts. They help to minimize errors associated with manual marking. Femotosecond laser capsulotomy marking method exploits the cyclotorsion compensating iris‐registered femtosecond laser–assisted capsulotomy marks as the axis reference [11]. This method reduces error due to surgical parallax and does not impact the strength or extensibility of the capsulotomy. Intraoperative aberrometry could assess the IOL axis alignment in real time during surgery, providing additional guidance for precise toric IOL placement [15].

However, the above marking methods are relatively complex and require certain equipment to implement, and manual marking methods are still used in most countries. Among manual marking methods, the advantages of the TRP method are obvious. In the labeling process, there is no need to consider the misalignments caused by the patient’s head position, eye position, and patient’s cooperation. We only need to randomly label three points in the corneal limbus and then use iTrace system or slit lamp photography with 360° Dial for measurement.

The current study is limited by the limited number of patients included. Nevertheless, the information provided in the current study is still important for both surgeons and clinicians to understand this new approach. Further studies with a larger number of eyes and longer follow‐up periods are needed to determine the benefit of the current approach. Another limitation of the current study is that it only used the digital image navigation system as the gold standard for comparison and obtained the axis alignment of the preoperative astigmatism axis. A prospective study is needed to obtain the refraction and visual acuity of patients with astigmatic IOL implantation. The third limitation is that this method relies on iTrace machines, but it is more cost‐effective than the digital image navigation systems and Femotosecond laser capsulotomy marking method. By incorporating the TRP method into clinical practice, medical professionals could enhance the precision and efficacy of the toric IOL implantation procedures.

## 5. Conclusion

The TRP marking is a safe and convenient method, which could be considered as an alternative method to determine the axis during toric IOL implantation. It could eliminate alignment caused by the surgeon’s own experience, as well as factors such as the patient’s pupil size.

## Author Contributions

Shuilian Chen: conceptualization, data curation, formal analysis, methodology, and writing the original draft; Yiliang Liu: conceptualization, data curation, supervision, and validation; Hongyang Zhang and Yongyi Niu: project administration, data curation, supervision, and validation; Fulong Luo: conceptualization, methodology, supervision, validation, and writing–review and editing; Yongyi Niu: writing–review and editing.

## Funding

This study was supported by the National Natural Science Foundation of China (82171036) from Hongyang Zhang, Natural Science Foundation of Guangdong Provincial (2023A1515011735) from Hongyang Zhang, National Natural Science Foundation of China (82501288) from Shuilian Chen, and Science and Technology Program of Guangzhou, China (2024A04J5045) from Shuilian Chen.

## Conflicts of Interest

The authors declare no conflicts of interest.

## Data Availability

The data that support the findings of this study are not publicly available because they contain information that could compromise the privacy of research participants but are available from the corresponding author upon reasonable request.
